# A review on *in silico* virtual screening methods in COVID-19 using anticancer drugs and other natural/chemical inhibitors

**DOI:** 10.37349/etat.2023.00177

**Published:** 2023-10-26

**Authors:** Babak Sokouti

**Affiliations:** National University of Singapore, Singapore; Biotechnology Research Center, Tabriz University of Medical Sciences, Tabriz 5165665813, Iran

**Keywords:** Virtual screening, coronavirus disease 2019, cancer, severe acute respiratory syndrome coronavirus 2, molecular dynamics, docking, repurposing

## Abstract

The present coronavirus disease 2019 (COVID-19) pandemic scenario has posed a difficulty for cancer treatment. Even under ideal conditions, malignancies like small cell lung cancer (SCLC) are challenging to treat because of their fast development and early metastases. The treatment of these patients must not be jeopardized, and they must be protected as much as possible from the continuous spread of the COVID-19 infection. Initially identified in December 2019 in Wuhan, China, the contagious coronavirus illness 2019 (COVID-19) is caused by the severe acute respiratory syndrome coronavirus 2 (SARS-CoV-2). Finding inhibitors against the druggable targets of SARS-CoV-2 has been a significant focus of research efforts across the globe. The primary motivation for using molecular modeling tools against SARS-CoV-2 was to identify candidates for use as therapeutic targets from a pharmacological database. In the published study, scientists used a combination of medication repurposing and virtual drug screening methodologies to target many structures of SARS-CoV-2. This virus plays an essential part in the maturation and replication of other viruses. In addition, the total binding free energy and molecular dynamics (MD) modeling findings showed that the dynamics of various medications and substances were stable; some of them have been tested experimentally against SARS-CoV-2. Different virtual screening (VS) methods have been discussed as potential means by which the evaluated medications that show strong binding to the active site might be repurposed for use against SARS-CoV-2.

## Introduction

Morbidity and fatality rates have skyrocketed because of the coronavirus disease 2019 (COVID-19) epidemic, making it a worldwide health emergency. New treatment medicines are desperately needed to combat this illness. As of the most current information available, which was on 26 April 2023, there have been a total of 764,474,387 confirmed cases documented around the globe, which has directly resulted in 6,915,286 fatalities (i.e., https://covid19.who.int/). It wasn’t until December 2019 that scientists confirmed the existence of severe acute respiratory syndrome coronavirus 2 (SARS-CoV-2) or coronavirus (CoV) illness, also known as COVID-19. CoVs are in the family Coronaviridae. They have a genome that is an enclosed, single-stranded, positive-sense RNA that ranges in length from 26 to 32 kilobases [1]. Humans are only known to be infected by alpha and beta CoVs, which fall into two of the four genera: alpha and beta [1, 2]. There is a 76.5% degree of similarity between the genomic sequences of SARS-CoV-2 and SARS-CoV. The RNA genome of the SARS-CoV-2 virus, which belongs to the family Coronaviridae and is positive-sense and single-stranded, is encased in a membrane envelope inside the virus. The Middle East respiratory syndrome coronavirus (MERS-CoV) and SARS-CoV viruses are members of the genus beta CoV, of which SARS-CoV-2 is a member. There are at least four structural proteins in SARS-CoV-2: the spike protein (S-protein), the envelope protein, the membrane protein, and the nucleocapsid protein [3]. The virus causes minor or no symptoms to death and is characterized by fever, dry cough, and fatigue [4].

The novel CoV (2019-nCoV) was initially named after its discoverer [5]. Angiotensin-converting enzyme two (ACE2) is the receptor for SARS-CoV-2 entry into epithelial cells lining the lower respiratory tract in recent studies [6–8]. Structural analysis and pharmacological experiments have shown that the spike of SARS-CoV2 interacts with human ACE2 receptors [9]. SARS-CoV-2 S1 S-protein interacts with ACE2 via its receptor binding domain (RBD), which then triggers membrane fusion mediated by the S2 domain and the incorporation of viral RNA into host cells. SARS-CoV-2 RBD binds to ACE2 through hydrogen bonds and salt bridges, resulting in a stable interaction with a dissociation constant [dissociation constant (Kd), in nano molar (nM)] [10].

CoV infection often results in a respiratory illness that is either mild or moderate in severity, and the majority of infected individuals recover entirely without requiring any medical treatment. COVID-19 infection, on the other hand, may cause severe respiratory distress in persons who already have preexisting diseases such as diabetes, respiratory disorders, cardiovascular difficulties, cancer, and so on, which require urgent treatment [11]. Due to their immunosuppression, cancer patients are more susceptible to these infections than others. According to research [12], people with cancer had roughly two times the risk of contracting COVID-19 compared to the general population. According to the findings of a research project that was carried out in Italy, it was discovered that among the 355 fatalities that COVID-19 caused, 20% of patients had aggressive cancer [13].

It is essential to identify highly conserved proteins across multiple CoVs as targets for antiviral therapy against SARS-CoV-2; since there is a significant possibility that CoVs may change to produce a new infectious virus. Many CoVs target enzymes involved in replication, such as RNA-dependent RNA polymerase (RdRp) and protease [6, 4]. Drugs that inhibit conserved proteases, such as main protease (Mpro) and papain-like protease (PLpro), can hinder viral replication and proliferation by interfering with the processing of essential viral polypeptides after they have been translated. This fact reduces the likelihood that mutations will lead to treatment resistance [14–16]. The CoV polyprotein encodes two proteases, a PLpro and a 3-C-like protease (Mpro). Non-structural proteins (Nsps) translated into cells are processed and secreted by combining two proteases (PLpro). SARS and MERS-CoV drug development targets included PLpro and Mpro proteases [17–20]. There is no cure for COVID-19; however, several therapeutic avenues are being investigated, and multiple clinical trials are underway [21–24].

There has been a great deal of research within herbal medicine in regard to anti-CoV agents since the outbreak of SARS. Among the most attractive targets are receptor and protein inhibitors. A number of compounds, including alkaloids (like tryptanthrin), flavonoids (like herbacetin), steroids (such as glycyrrhizic), tannins (such as pedunculagin), quinones (such as emodin), and stilbenes (such as curcumin), inhibit SARS-CoV-2 replication and transcription, and may therefore be of value in early virus cycles [25]. Many research groups were forced to seek out new synthetic molecules that would be effective against SARS-CoV-2 due to the COVID-19 pandemic. By expressing the 3-chymotrypsin-like protease (3CLpro) protein, the virus is capable of proteolyzing nonfunctional viral proteins into functional ones. A novel series of fused 1,2,3-triazole derivatives has been shown to inhibit 3CLpro protein [25]. Several synthetic drugs have shown the ability to inhibit CoV infection *in vitro*. During viral entry, MDL28170, trypsin, and leupeptin inhibit the activity of endosomal proteases [26], preventing the spread of infection. In the study, ribavirin, penciclovir, favipiravir, nafamostat, nitazoxanide, remdesivir, and chloroquine were found to be the most effective drugs against SARS-CoV-2. Hydroxychloroquine has been tested for its effectiveness in blocking virus infection in a number of clinical trials [27]. COVID-19 therapeutic strategies could, however, be improved through antibodies. Many patents are currently being examined to determine whether antibodies against COVID-19 can improve therapeutic outcomes [25]. Plasma from patients who have been cured can be used to treat patients with COVID-19. A clinical vaccine trial uses antigens of SARS-CoV-2 to direct the immune system against these antigens and lower COVID-19 complications. There have only been some vaccine trials that have reached the third stage of clinical development [25].

The urgent need to address COVID-19 is not met by the lengthy *de novo* medication development procedure [28]. Finding new medications based on the repurposing strategy is the most pressing option for combating COVID-19 [3]. These results led to the use of X-ray crystallography and structural analyses of the SARS-CoV-2 RdRp to develop novel antiviral drug designs and drug repurposing techniques [29, 30]. Attempts to find novel treatment agents for COVID-19 via drug repurposing hold much promise. As part of therapeutic repurposing, current medications are tested for their potential to block the viral protease that catalyzes the cleavage of the viral S-protein. This strategy has the benefit of being able to move quickly since a significant number of the medications have previously undergone clinical testing and are, as a result, recognized to be safe for use in people.

**Figure 1:**
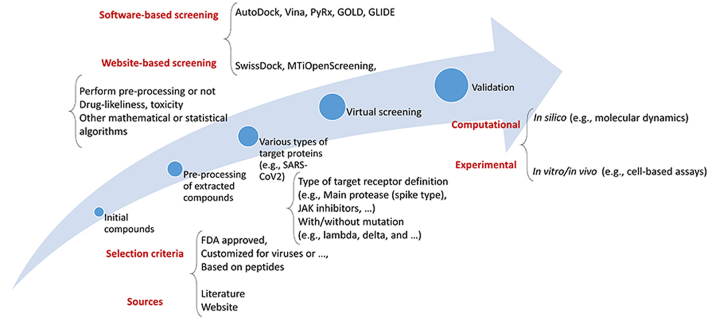
A general flowchart for the VS approach. JAK: Janus kinase

As such, a thorough virtual screening (VS) investigation was conducted using a library of Food and Drug Administration (FDA)-approved medications to identify compounds capable of binding to the three primary molecular targets of SARS-CoV-2 [Protein Data Bank (PDB)] [31, 32]. Because of its utility in the quest for novel therapies for COVID-19, VS has become a standard computational tool in drug development. Using computational approaches, VS evaluates a pool of therapeutic options. This strategy is fast and effective, allowing for the rapid screening of many molecules. A routinely used flowchart for the VS process is shown in Figure 1.

When looking for novel treatment drugs to treat COVID-19, adopting VS may be pretty beneficial due to its numerous benefits. The use of VS allows for the screening of vast numbers of compounds quickly and effectively. Moreover, VS may be used to find compounds structurally related to existing medications. The potential benefits of this method include the speed with which promising medication candidates may be found. The use of a trustworthy computational technique to reliably anticipate binding free energies (BFE) is crucial for the success of any VS attempt [33]. VS has a poor success rate and predicts primarily false positives. The authors successfully designed a system for VS that considerably cuts the number of false positives by combining two types of filtering: pre-docking filtering, which is based on form similarity, and post-docking filtering, which is based on interaction similarity [16]. VS of current drugs is often used as a first step in repurposing them to treat emerging disorders like COVID-19; this is then followed by experimental validation. However, the actual hit rate is generally relatively low when using standard computer methodologies [33]. Moreover, various epidemiological and systems biology-based researchers have been in this field [34–36].

This article will explore how VS may be used to find prospective medication candidates for COVID-19. VS concepts will be discussed initially. After that, we’ll explain how we plan to utilize VS to identify potential novel COVID-19 treatments.

## Cancer and COVID-19

Antiviral cytotoxic T (CD8+ T) lymphocytes cell responses are triggered by SARS-CoV-2 and then directed towards tumor cells based on their cancer-specific epitopes. The potential therapeutic advantages of repurposing antiviral CD8+ T cells against malignancy are enormous. In anticancer immunotherapy, CD8+ anti-tumour T cells are generated in response to auto-immunogenic epitopes strongly present in tumor antigens. It is possible to show synthetic viral epitopes on the surface of cancer cells to reprogram CD8+ T lymphocytes, which would typically target and destroy viruses, to target and destroy tumors instead. For effective cancer immunotherapy, CD8+ T cells are crucial. It’s possible to redirect CD8+ T lymphocytes toward tumors by displaying synthetic viral epitopes on the surface of cancer cells [37].

Interferons significantly impact various immune responses, including antiviral responses, adaptive immunological responses, and fungal and viral immune responses. A little window of opportunity exists for treating COVID-19 with anti-interleukin-based drugs; some patients may have different problems. For the treatment of severe ARDS brought on by COVID-19, the experimental data point to the potential benefit of tocilizumab and selinexor. The immunological response to COVID-19 in humans may be modulated by the monoclonal antibody C-C chemokine receptor type five (CCR5; immune and non-immune). Anti-interleukin-6 (IL-6) monoclonal antibodies may mitigate COVID-19’s inflammatory storm. JAK inhibitors, leronlimab, and Bacillus Calmette–Guérin (BCG) are a few of the medications that researchers are testing to treat COVID-19. Lenalidomide, a drug often used in treating multiple myeloma, is also being investigated for its potential use in treating COVID-19 in elderly patients with mild to moderate symptoms [38].

Recent studies, including docking and simulation methods, employed the replication delay protein (RdRp) from SARS-CoV-2. The CASTp 3.0 program used computational geometry techniques to identify the active site of SARS-CoV-2-RdRp- associated nucleotidyl transferase domain (NiRAN). Sixteen medications were validated by AutoDock docking to the Computed Atlas of Surface Topography of Proteins (CASTp) predicted active site of the viral domain. Molecular mechanics with generalised born and surface area (MM-GBSA) solvation was applied to the compounds that had the most incredible docking findings. Using a default configuration of molecular dynamics (MD) software (i.e., Desmond), the authors simulated the stability of the SARS-CoV-2-RdRp-NiRAN domain-ligand complex [39].

To dock 27 drugs commonly used to treat lung cancer, Pingali et al. [40] opted for LigPrep. Molecular docking and calculations of binding affinity were used to locate the appropriate ligand for the proteins that were being targeted. Ten ligands with a docking score of 4.0 were selected for additional screening and evaluation against the RdRP. The MD simulation was conducted in Groningen MAchine for Chemical Simulation (GROMACS) 2018.2 using the charmm36-mar2019.ff force field and all-atom optimized potentials for liquid simulations (OPLS-AA) for protein. The number of Hydrogen Bond interactions, Radius of Gyration, and root mean square deviation of the structural order were calculated using GROMACS software in a MD study.

A Lamivudine docking investigation was performed utilizing the recently released cryo-EM structure of SARS-CoV-2-RdRp. Lamivudine may prevent cytidine triphosphate (CTP) from catalyzing SARS-CoV-2-RdRp RNA Polymerase by inhibiting chain termination. Neither monkey nor human malignant cells showed any inhibition of SARS-CoV-2 replication when treated with Lamivudine; however, it may help treat or prevent COVID-19.

On the other hand, antiretroviral nucleoside/nucleotide analogs (ANNAs) were made to stop viruses like Ebola and human immunodeficiency virus (HIV) from making RdRp. Still, it has been found that they can be used against a different group of viruses. Lamivudine has been given to HIV/hepatitis C virus (HCV) patients for over 25 years. Even though it has been used a lot, it hasn’t been linked to too many severe side effects, which makes it a safe drug that can be used alone or with other antivirals. Since viruses cause certain human malignancies, the concept that ANNAs may treat cancer is not new. Remdesivir is another pro-drug that attaches to the Severe Acute Respiratory Syndrome (SARS) virus. However, it does so with a lower affinity than lamivudine [41].

Hyaluronic acid (HA)-based nanoparticles are uptaken more readily with doxorubicin (DOX) in radiation therapy, drug resistance is overcome, and side effects are reduced [42]. Furthermore, smart HA-based delivery systems are capable of developing new drugs as well as responding to internal and external stimuli. In addition to DOX and other antitumor compounds, several types of gene therapies based on RNA or DNA can enhance their effectiveness. HA-based delivery systems are effective in delivering drugs or genes with DOX [42].

In addition to controlling proliferation and migration rates, exosomes modulate the tumor microenvironment and are widely recognized as key players in cancer progression and therapy. The use of exosomes as carriers of genetic tools and antitumor agents is an important component of controlling the progression of cancer [43].

P-glycoproteins (P-gp) are to blame for DOX resistance since they transport the medication. The presence of dox resistance may be determined by investigating the molecular pathways that control P-gp. These pathways include nuclear factor erythroid 2-related factor 2 (Nrf2), hypoxia-inducible factor 1-alpha (HIF-1a), microRNAs (miRNAs), and long noncoding RNAs (LncRNAs). Since DOX’s anticancer action is improved when adenosine triphosphate (ATP) is low, it seems sense that P-gp activity is hindered when ATP levels drop. The delivery of DOX together with anticancer drugs and genes that enhance DOX’s cytotoxicity may be accomplished by the use of nanoarchitectures like liposomes, micelles, polymeric nanoparticles, and solid lipid nanocarriers. And again as mentioned before, the HA can be beneficial to the surfaces of nanocarriers for improving the overall performance considering the long run [44].

## Drug repurposing procedures based on computational aspects

### Data sources and tools

A summary of the database or data sources along with those commonly applied tools by researchers whether they are standalone, packages of software environments, or web services is listed in Table 1 demonstrating their functionality in different parts of the VS procedure.

**Table 1:** List of data sources and tools useful in VS approaches applicable to COVID-19

**Database and resources**	**Pre-treatment tools**	**Docking tools**	**MD tools**	**Analytics Platforms**
DUD-E web server (useful docking decoys)	OpenBabel/Pybel (package for creating or converting different ligand/protein types)	PyRx (python package) for VS of libraries of compounds against potential drug targets	GROMACS	KNIME (broad range of free/commercial packages for analysis)
SWEETLEAD (database of curated chemicals)	ProTox (virtual lab for the prediction of toxicities of small molecules)	AutoDock Vina (C++/C)	AMBER, a suite of biomolecular simulation programs, (MM-PBSA and MM-GBSA for clusters/GPUs)	Molinspiration (broad range of cheminformatics software tools)
ChEMBL (curated database of bioactive molecules with drug-like properties)	LigandScout (generates 3D pharmacophore models for VS)	Autodock (C++/C)	SimpleRun (equilibrate protein-ligand complex and produce MD simulation)	PlayMolecule (one-clink molecular discovery)
SuperDRUG2 (resource for approved/marketed drugs)	PlayMolecule (preparation of protein for MD simulations)	rDock (ANSI C++)	Desmond from Schrödinger	ChemBioServer 2.0 (filtering, clustering, and VS)
DrugBank (information on drugs and drug targets)	LigPrep from Schrödinger	YASARA (AutoDock/Autodock Vina)	Schrodinger’s MD	Weka Software (Data mining: practical machine learning tools and techniques)
GenBank (NIH genetic sequence database)	MetaPocket: (improve protein ligand binding site prediction)	GOLD (genetic optimization for ligand docking)	YASARA (GROMACS module)	ChemoInfo (cheminformatic tools and databases for pharmacology)
LOPAC (library of pharmacologically active compounds)	Pockdrug server (druggability prediction)	MTiOpenScreen online webserver for VS	-	-
UniProt (database of FASTA protein sequences and functional information)	AMMOS2 web service (refinement of protein–small organic molecule complexes)	iDock (VS tool)	-	-
PubChem (large database of chemicals)	BindingDB (information on properties of ligands/targets)	FireDock (scoring of protein–protein docking solutions)	-	-
HOMSTRAD (HOMologous STRucture Alignment Database) is a curated database of structure-based alignments for homologous protein families	ReScore (+ scoring docked positions)	MVD	-	-
-	MedChemExpress (MCE) includes high quality inhibitors and recombinant proteins	-	-	-
-	ASINEX’s BioDesign (with key structural features of known pharmacologically relevant natural)	-	-	-
-	ACPYPE (or AnteChamber PYthon Parser interfacE), a ANTECHAMBER wrapper script for the generation of small molecule topologies and parameters	-	-	-

DUD-E: Directory of Useful Decoys-Enhanced; MM-PBSA: molecular mechanics Poisson-Boltzmann surface area; MVD: Molegro Virtual Docker; 3D: three-dimensional; -: blank cell

### Literature information

Ebrahimi and coworkers tested 55 antiviral agents in their simplified molecular-input line-entry system (SMILES) and structured data file (SDF) formats. The SARS-CoV-2 spike receptor-binding domain, in conjunction with acetyl-CoA cyclase (ACE2), was obtained as a 3D crystal structure using the PDB website. S-protein’s S1 subunit underwent pre-processing to eliminate extra water molecules and irregular residues. And, PyRx docking was used to place 242 compounds that had passed the first VS phase onto four different receptors. Molecular docking was employed to assess binding affinities, and the AutoDock Vina program was used for this purpose.

Molecular docking and MD simulation were used to conduct an *in silico* investigation. Decoy chemicals identical to nelfinavir were obtained from the DUD-E web server to verify the docking parameters. GROMACS 2016, a biomolecular software tool, was used to run MD simulations on protein-ligand complexes found in the optimum posture of molecular docking. A sufficient quantity of sodium and chloride ions were introduced into the systems to bring about the desired neutralizing effect. Two 500-ps equilibration phases in constant number, volume, and temperature (NVT) and constant number, pressure, and temperature (NPT) ensembles produced the reduced systems after the steepest descent and conjugate gradient energy minimization. Eight MD simulations were conducted to compare the effects of ATV and PRZ, as well as an inhibitor of the viral S-protein called K22 [45].

In an investigation, the S-protein of the SARS-CoV-2 virus was modeled using the GROMOS54a7 force field and the SPC model for water as the solvent. Periodic boundary conditions maintained constant pressure and temperature, and long-range electrostatic forces were accounted for with the help of the smooth Particle-Mesh Ewald (PME) technique. The SARS-CoV-2 viral S-MD protein’s equilibrated structure was used for docking calculations. VSs were performed using the AutoDockTools program and the AutoDock Vina technique. The SWEETLEAD library was searched for ligands since it was constructed from pharmaceuticals included on many official medication lists. As a follow-up to docking calculations, MD simulations were run on the S-protein complex with the top three ligands. Following an equilibrated MD simulation that lasted for 18 nanoseconds, the MM-PBSA method was used to calculate the binding free energy for each complex [46].

A docking study optimizing the Mpro homodimer structure in HMHC and calculated energy under the AMBER99 force field was conducted by Tsuji et al. [47]. With the help of rDock, they could run docking simulations on a 3D structure database including 1,485,144 molecules. Information on 27,561 unique compounds was gathered using KNIME version 4.1.2 and their corresponding ChEMBL (identifications) IDs. After running docking simulations using AutoDock Vina, 513,597 docking modes were found, all eliminated using a cutoff score of –10 kcal/mol [47].

Moreover, some anti-HIV medications, such as sildenafil (used to treat leprosy, erectile dysfunction, and pulmonary hypertension) and thalidomide (used to treat multiple myeloma), have been successfully repurposed for use against 3CLpro. Under normal conditions, the researchers used high-throughput VS to identify protein-binding sites. The descriptions of the detected binding sites included information on hydrogen bonds, exposure and enclosure, size, connection site points, tightness, and the nature of either hydrophobic or hydrophilic molecules. The molinspiration tool was utilized after three rounds of VS of anti-HIV and traditional Chinese medicine (TCM) drug databases to forecast bioactivity outcomes. In all, a two fs time step was used to run an MD simulation that lasted 100 ns. In the script MM-PBSA.PY and using the Amber18 program, the authors found that there are two stages to calculating the binding free energy of protein-ligand complexes: lowering the energy, warming it up, and equilibrating [48].

An energy-optimized pharmacophore hypothesis was proposed based on the crystal structure of SARS-CoV-2 Mpro in association with the non-covalent inhibitor X77. Four thousand six hundred medicines from the SuperDRUG2 database were used in a VS process based on e-pharmacophores. At a nearly neutral potential hydrogen (pH), the ligands’ structures were optimized. One thousand medicines were screened using the e-pharmacophore model to see whether they interacted with the active site of Mpro. A structure-based VS was carried out using molecular docking to rank the medication according to its binding affinities. Using the prime module and MM-GBSA approach in Maestro 11.4, the BFE of 40 medicines were determined. The researchers used the Desmond module of Schrodinger’s MD Simulator to run MD simulations to examine the drug- Mpro binding’s resilience in an explicit solvent environment. MM-GBSA was used to determine each complex’s binding energy after the MD simulation [49].

Likewise, Rahman et al. [50] employed the PyRx VS techniques to conduct molecular docking based on the 3D crystal structure of SARS-major CoV-2 retrieved from the PDB database. To further refine the best 31 drug candidates, researchers used the semi-empirical PM6 optimization method implemented in Gaussian 09 and performed further docking using AutoDock Vina and GOLD. To investigate the potential for conformational changes and interactions between the apo and holo forms of 6LU7 over 100 ns, MD simulations were run on the molecule. To record the backbone, alpha carbon, and heavy atoms, YASARA was used to examine the results of 100 ns MD simulations. The binding free energy was calculated using the AMBER14 force field and the MM-PBSA method in YASARA dynamics software. Principal component analysis (PCA) was used to compare and contrast the energy profiles generated from the MD trajectory data [50].

The pharmacophore model was developed and validated using data from 166 compounds. Recent researchers looked at 144 DUD-E-produced decoys, 154 inactive molecules, and 12 SARS-CoV Mpro inhibitors found in the literature. The sensitivity, specificity, and enrichment factors were used to measure how well the model worked. Using a genetic algorithm based on GOLD version 5.8, the authors docked molecules onto the crystal structure of SARS-CoV-2 Mpro in association with the reversible inhibitor “X77”. Fifty complexes per chemical were constructed using the complete linkage method, and then root mean square deviation (RMSD)-based clustering was utilized to classify them. The accuracy of the pose predictions was measured by calculating the RMSD of the heavy atoms between the docked poses and the original PDB coordinates of X77. Utilizing a validated pharmacophore model, GOLD was used to screen 2,684 FDA-approved medications for activity against SARS-CoV-2 Mpro. Select protein-drug complexes were subjected to MD simulations to gain insight into the structural stability of these complexes over the long term. VS yielded six promising hits, and MD simulations using the SARS-CoV-2 Mpro were used to model these hits. AMBER ff14SB, an atomic-level force field, and Ambertools 14 were used to generate topology and coordinate files. MD simulations were performed using the AMBER MD program PMEMD.cuda, using 306 residues, 20 ps for heating, 50 ps for density equilibration, and 150 ns for production [2].

Collecting various chemicals that can fight nine different lethal CoVs was used to extract the attributes of tiny compounds and protein targets. After that, classification algorithms were used to assess the possible association between all active drugs that fight pathogenic CoVs and their targets. Ten rounds of cross-validation were performed once drug-target combinations were generated. Thirty-nine pairings of active compounds tested against COVID-19 and drugs authorized by the FDA were included in the dataset that was used to evaluate each model’s capacity to generalize. Using these six metrics, the authors evaluated the relative strengths of the two systems for target prediction and VS. Foreseeing the targets required computing the Euclidean distance that separated the molecule from each target in the multimodal common space [51].

Docking ligands were created in Pybel and optimized using the MMFF94 force field, and the FDA-approved medications were acquired from the DrugBank database. SARS-CoV-2 target protein structures were collected from the PDB. They also used ProTox to analyze the potential toxicity of potential medication candidates. Protein-ligand docking study of SARS-CoV-2 target proteins was conducted using the molecular docking program AutoDock Vina. Two target proteins were utilized to demonstrate the selection process and how drug screening technology generalized to other proteins. Candidates with low docking scores on both proteins were prioritized. To account for the fact that docking scores are highly dependent on the structure of the target molecule, the top 40 hits were selected for each protein based on docking scores and then looked for medicines that were shared by several proteins [52].

The GenBank entry annotation was mined by Chen et al. [53] for the SARS-CoV-2 polyprotein (PP1AB) sequence, which was then compared to the SARS-CoV PP1AB sequence. Following the high-resolution apo-enzyme structure of SARS-CoV 3CLpro (PDB ID: 2DUC) as a template, GROMACS 2018 was used to perform the steepest descent energy minimization on the SARS-CoV-2 enzyme structure. The screening was performed using the MTiOpenScreen online service, which offers a library of 7,173 commercially available pharmaceuticals consisting of 4,574 compounds and stereoisomers. The active-site residues dictated the grid center of the target binding site. The best ten scores from each chain were shown when MTiOpenScreen yielded 4,500 target-ligand docking combinations [53].

The GROMOS96 43a1 force field from the GROMACS 5.1.1 suite was employed on a macOS Mojave workstation with an 8-core Intel Xeon E5 center processing unit (CPU). Using SWISS-MODEL, researchers were able to homologize the S1-subunit of SARS-CoV-2 and, following the lead of the S-protein crystal structure, construct a 3D model of the anti-S-protein receptor binding domain (S-RBD) protein. Using a computational screen, the authors identified compounds able to bind S-RBD. The LOPAC drug library was used to find potential antiviral treatments. Possible therapeutic candidates were docked into ACE2 crystal structures after structural modeling of the S-RBD of SARS-CoV-2. The ACE2 receptor protein crystal structure was employed with the SARS-CoV-2 model to determine the structure of the S-RBD S-protein. Through AutoDock Vina and PyRx, the authors checked the LOPAC library for molecules that bind to ACE2 and S-RBD of SARS-CoV-2. MD simulations were run on the ACE2 protein and the S-RBD protein using the GROMOS96 43a1 force field [54].

In another investigation, the authors assembled an approximately 8,000-molecule library of chemicals from medication databases validated by various authorities. There were three primary collections from Drugbank, FDA, and ZincWorld. Three ligands, 6W63, 5R83, and 4MDS, were taken from their corresponding complexes in the PDB, and a fourth, C10, was designated for use in ligand-based approaches. The structure-based VS using docking approaches has been verified via the help of redocking as well as a receiver operating characteristic (ROC) curve analysis. Three Mpro structures were chosen and constructed from the PDB and used in a validation method utilizing the GOLD program to assess the success of this docking strategy in recovering known active compounds from a dataset of actives polluted with ‘decoys’. For each query, the authors extracted the top 100 compounds based on their scores, analyzed their toxicity profiles, and then put the survivors through docking to look at how they interacted with the enzymes in question [55].

The researchers extracted required data from the DrugBank and FDA databases to identify potential repositioned medications. More than 1,800 different medications and over 9,000 drugs in various stages of clinical testing and experimental research are included in these databases. Using the PDB protein database as a starting point, a 3D structure-based pharmacophore model was constructed to locate the inhibitor N3 (7BQY) binding site. 3D positions of the minor possible molecular interactions were calculated using the pharmacophore model. LigandScout 4.4 was used for a pharmacophore model-based VS. The winning molecules were identified by averaging the most outstanding affinity values from docking poses calculated using the Autodock Vina 1.1 extension set and a visual assessment of the compound docking postures. With the MMFF94s force field, they optimized the energy of each compound by adjusting the atomic charges and the potential energy. The generalized Born solvation model was utilized to calculate the overall energy and induced-fit docking score. ChimeraX was used to visualize the docking data, and a consensus score was used to choose which molecular hits to pursue [56].

It was found that the AMBER99 force field was the most accurate in calculating the energy of an RdRp structure. rDock, a highly recognized and speedy docking simulation software, was used to conduct simulations on a compound library after removing favipiravir-RTP. The 249 medicines discovered by AutoDock Vina docking simulations were investigated using these techniques. The docking modes were narrowed down by utilizing the scoring criterion of 12 kcal/mol that AutoDock Vina provided. Single-point calculations were used to calculate the energies of interactions between fragments [57].

Potential biological activities and molecular properties of drug candidates were determined using Molinspiration’s VS engine, MiScreen, and Molinspiration’s property engine. Using the CHARMM- graphical user interface (GUI) web interface, amino acid partial charges, bond orders, missing atoms, and side chains were calculated using the PDB crystal structures of the selected targets. Nevertheless, the whole 3D architecture of viruses cannot yet be accessed. After retrieving the corresponding FASTA sequences from UniProt, the authors built a homology model using the SWISS-MODEL service. Nine medicines [Bruton tyrosine kinase (BTK) inhibitors] were retrieved from the DrugBank database, and their 3D structures were compared. During structural refinement, PyRx v0.8 and the unified force field were applied. Using AutoDock Vina inside PyRx, the authors digitally screened the structures of the selected medicines. It was determined that ACE2, BTK, membrane protein, Mpro, nucleocapsid phosphoprotein, Nsp14, PLpro, RdRp, SRBD, and TMPRSS2-specific dimensions would be used to dock the virus and host proteins in a grid box. As the drug with the highest binding energy was used in Extra-precision (XP) precise docking, the states with the least total energy were chosen. A CHARMM36 force field was used for MD simulations, which ultimately used nanoscale MD to simulate target-ligand complexes based on their Glide docking scores [58].

Using Clustal Omega and the UGENE platform, the whole genomes of 111 SARS-CoV-2 samples were lined up. With the help of Modeler, researchers could make educated guesses as to the shape of Mpro in its functional state. The GROMOS approach categorizes conformers generated by a GROMACS MD simulation. Molecules having the ability to inhibit Mpro were identified by docking them into their active site using iDock. The researchers investigated the top 10 molecules in each conformer to find Mpro inhibitors [59].

From the database of 2,683 drugs authorized by the FDA, 7.2 million 3D protomers were downloaded and screened for instability and high activity. The researchers managed two different virtual displays with the assistance of MOE 2019 and Autodock Vina. MOE-based virtual screens used AMBER14:EHT force fields. MOE 2019 prepared the SARS-CoV-2 3CLpro crystal structure by 3D protonation and docking. All of the top 50 postures were submitted to Induced Fit to undergo further development and reduce their energy consumption. Pharmacophores were built and searched using the top-scoring ligands from 3CLpro complexes. By first identifying the optimal binding sites between a protein and its ligand, structure-based drug design may insert beneficial substituents to serve as hydrogen-bond acceptors and donors of pocket amino acids [60].

Using a mapping algorithm, the authors of this study converted the amino acid sequences of the target proteins from the SARS-CoV virus into the sequences of the equivalent proteins from SARS-CoV-2 to generate 3D homology models. The research comprised 1,577 medicines, 39,442 natural items, and 115 herbal supplements. After determining which chemicals had the lowest binding energies, docking simulations were run on them. In this case, supervised machine learning was used to identify compounds with therapeutic potential against SARS-CoV-2. MD were simulated using PlayMolecule. The MD simulation was done with SimpleRun after the ligand was set up and the binding domain for the S-protein receptor was built [61].

To determine binding sites, Kumar et al. [62] isolated the RBD from the ACE2-RBD complex crystal structure and put it through the protein preparation wizard. One thousand-one conformation of the spike RBD was clustered using the Desmond trajectory clustering tool, with hierarchical clustering using average linkage as the clustering method of choice. Multiple 3D conformations of approved drugs were generated using the LigPrep module, which was used to download the set of approved drugs from the drug bank. Using a grid-generating program, a grid was developed around the RBD crystal structure’s expected binding location. They ran 200 ns MD simulations using Desmond on each hand-picked RBD-drug combination. By averaging MM-GBSA energies, it was possible to compute conformational changes and fluctuations and quantify binding strength [62].

Additionally, the active site of PLpro (PDB structure 6W9C protein) was predicted using a meta server called MetaPocket 2.0 with a threshold of five pockets. The similarity threshold (Tc 0.75) was used to identify compounds with high Tc scores from the large chemical universe, and the compounds were ranked based on their Tc scores’ similarity to the reference PubChem COVID-19 clinical trial chemicals. Six different mathematical models were employed to predict the chemical absorption, distribution, metabolism, excretion, and toxicity (ADMET) properties of the selected hit compounds accurately. A collection of molecules from the FDA-approved medications, PubChem COVID-19 clinical trial chemicals, NPASS, and Maybridge databases were molecularly docked by the researchers using AutoDock Tools (ADTs) 4.6. Each compound that was put through the screening process had ten different configurations. Clustering was performed on the docked virtual compounds using the Hierarchical Clustering technique and the online ChemBioServer tool. MD simulations were run in GROMACS-4.6.5, ligand topologies were made in PRO-DRUG, and the tangled structure was dissolved in water. Ligands’ BFE to proteins were determined using a MM-PBSA that incorporates molecular mechanics energies. The BFE is crucial to finding new medicines [63].

The second group of researchers also assembled 29 antiviral drugs in SDF (3D) format from the PubChem database. These antiviral medicines include ones that have been used in the past to combat a variety of viruses. Molecular docking was used to examine the binding affinity of 39 antiviral drugs with different SARS-CoV-2 proteins and protein domains. Docking of protein molecules was accomplished with the help of the FireDock refinement tool and an algorithm based on the shape-based complementary idea of docking. Swiss-Similarity’s online application was used to rank the prospective therapeutic compounds against SARS-CoV-2. Swiss Target Prediction’s web application was used to forecast the most probable macromolecular targets of the best medications [64].

Mahdian et al. [30] used the CD147 protein and the ACE2-SARS-CoV-2 RBD complex to set up the system input and create the first structures at a resolution of 2.8 angstroms. In this work, the VS was done using the PyRx tool, docking was done on the Pockdrug server, and drug discovery was made with Autodock Vina. Using Biovia Discovery Studio and MVD, they could see the eight postures that were saved at the end of the docking run. Maximum free binding energy (G = –10 kcal/mol) was attained with five medications bound to ACE2, four drugs bound to RdRp, and 7 drugs bound to CD147. During the MD simulations, a short-range electrostatic interaction was employed, and a distance restriction of 1.2 nm was applied to the van der Waals contact. Using the MM-PBSA approach and steepest descent algorithm, the binding free energy was computed [30].

Using a molecular docking strategy based on AutoDock Vina and the MTiOpenScreen Web service Teralı et al. [65] virtually assessed all Drugs-lib ligands against the receptor. The AMMOS2 web service was used to optimize further and rescore the effective docking ligands [65].

The S1 S-protein was the focus of the investigation of Prajapat’s et al [10]. Since PDB ID 6M17 showed the whole S1 protein RBD as well as the details of the interaction between the S1 RBD and ACE2, it was taken into consideration. To determine whether or not the crystal structure of the protein of interest is accurate, the PROCHECK program hosted on the SAVES v5.0 server was used. The ligand-receptor target binding free energy was calculated using Prime MM-GBSA and VSGB solution within 5.0. The efficiency of the VS technique was evaluated using enrichment experiments employing ROC and Boltzmann-enhanced discrimination of ROC (BEDROC) measurements. Desmond module was used to model all of the ligand-target complexes for 50 ns, and apo forms and S1 RBD-drug complexes were simulated using MD [10].

In a study, the mined data for Mpro sequences from the NCBI GenBank or GISAID (https://www.gisaid.org/) was employed by Kandeel and Al-Nazawi [20]. Sequences of SARS CoV and MERS CoV were retrieved from the GenBank database. Multiple sequence comparisons and Mpro sequence alignment were performed using the CLC genomics program. Once all compounds had been entered into Ligprep software and translated, the OPLS2005 force field was utilized for 3D optimization. In a computer simulation of the screening procedure, the COVID-19 Mpro crystal structure (PDB ID: 6lu7) was used for docking. Researchers found that curcumin significantly inhibited SARS Mpro [20].

In a recent study, after employing ADMET filters, the top 100 results from two SARS-CoV-2 Targeted Libraries were narrowed down to the ten most promising compounds. The GOLD 2020.1 program was used for molecular docking simulations. Tanimoto coefficients (Tcs), which measure structural similarity between molecules, are available on the BindingDB webpage. This information allowed isolating a class of molecules sharing an activity level greater than or equal to 40% [66].

For verification, each of the inhibitors docked during the initial step was docked into the substrate-binding aperture of each of the three SAR-CoV-2 3CLpro structures. AMBER20 and Gaussian09 MD simulations examined lapatinib, a potent SARS-CoV-2 3CLpro inhibitor. For the purpose of extracting pharmacophore characteristics from the last one hundred nanoseconds of MD trajectories of the lapatinib/SARS-CoV-2 3CLpro complex, the LigandScout software and the KNIME analysis platform were used. In every test, 0.2 M of 3CLpro was utilized as the concentration. The enzyme activity was evaluated with and without a 100 m inhibitor present [67].

The PDB (7BQY) of the SARS-CoV-2 Mpro enzyme’s crystal and 3D structure was used in the all-purpose molecular modeling program TINKER to conduct a systematic conformer search on all ligands by Mazzini et al. [68].The semi-empirical quantum chemistry software MOPAC7 Baker’s Eigen Following approach was used to refine the lowest energy conformation of each ligand. Researchers optimized the geometrical characteristics of the ligands by using the AutoDock Toolkit and the SARS-CoV-2 Mpro model. The AddSol tool was then used to include the improved structures in the overall complex. The blind docking option in AutoDock Vina was used to conduct these VS trials. They used the GROMACS 2018 program to do MD simulations to investigate the systems’ robustness. Under the NPT condition, they produced dynamical states with no constraints for 1.0 ns and took pictures at one ps intervals to see the results [68].

As a result of conducting a VS on a repurposing library that contained different pharmaceuticals, some compounds were found to be effective CoV inhibitors. The OPLS3 force and energy minimization program was used to generate other ligands by counting ambiguous chiral centers. Docking of the monomer A of SARS-CoV-2 3CLpro with the N3 inhibitor was performed. The docking search resulted in the elimination of inactive compounds from the pool of potential matches. FRED gives each docked position a score to calculate the potential shape field, and then it uses the poses with the highest scores to calculate the density fields. To accomplish protein reduction, both the OPLS3 force field and the Glide5.0 software were utilized. All the poses generated by the four different docking algorithms were rescored by ReScore + [69].

A RED function-based accelerated free energy perturbation-absolute binding free energy (FEP-ABFE) prediction method was employed for the VS of SARS-CoV-2 Mpro inhibitors. During the first molecular docking stage of the VS, the crystal structure of SARS-CoV-2 Mpro (the virus responsible for COVID-19) was used. The best 100 ligands found by molecular docking were then subjected to further evaluation using speeded-up FEP-ABFE simulations. The IC50 was calculated by adding a drug at six different concentrations to a well with 20 m of the substrate and 500 nM of the enzyme. Fifty liters of substrate were used to initiate the process [33].

At a resolution of 1.95, the crystal structure of SARS-CoV-2 Mpro bound to a ketoamide inhibitor was determined. Two residues were grafted from another Mpro crystal structure to fill in the gaps. Cryo-electron microscopy (EM) and MD simulations (using the GROMACS 4.5 package) were used to solve the structure of RdRp of SARS-CoV-2 at 2.90 resolution. Structural cluster analysis was used to choose one of the many possible population structures with the lowest local energy. It allowed for further refinement of the RdRp structure. This technique is possible because of the known co-crystal structure of the RdRp-NTP protein in SARS-CoV-2. Compound libraries were built using 6,218 FDA-approved and clinical trial medications, then standardized, and salts eliminated, all with the help of the rational discovery kit’s (RDK’s) MolVS module. The authors performed pre-docking filtering with shape similarity using active ligands for Mpro and RdRp derived from SARS-CoV-2 or other virus co-crystal structures. More specifically, AutoDock Vina determined the degree of binding affinity between 6,218 drugs currently in use or clinical trials and their respective target proteins. MedChemExpress supplied them with Remdesivir, Blonanserin, Emodin, Hypericin, Omipalisib, and Tipifarnib. Mpro and RdRp tests were performed in their lab using a 3CL protease, untagged (SARS-CoV-2) assay kit with an EnsightTM Multimode Microplate Reader. Binding free energy studies were performed on protein-ligand complexes built using docking simulations. To raise the temperature of the systems contained in a dodecahedron box filled with TIP3P water up to 300 K, the NVT ensemble was used. The free energy of binding was calculated from the three ns MD trajectory using MM-PBSA [16].

While the [Protein Data Bank, Partial Charge (Q), & Atom Type (T)] pdbqt format is required for use with Autodock Vina, SMINA may read in receptor files in either the PDB or pdbqt formats. The medication approval list and structures were retrieved from the SuperDRUG2 database. Molecular docking research was conducted using Autodock Vina and SMINA, and a VS pipeline was established with the help of Obabel, Pymol, and Rasmol. While SMINA was utilized for docking calculations, including stiff and flexible active site residues, Autodock Vina was used for those with rigid residues. For H41, C145, M49, and Q189 docking in the active site of SARS-CoV-2 Mpro, SMINA was utilized because of its flexibility. Due to the presence of flexible residues in the active site, SMINA predicts more remarkable binding affinities in most instances [70].

Structures of other SARS-CoV/ACE2 complexes were evaluated for structural alignment, and molecular docking was performed using the crystal structure of the RBD domain of the SARS-CoV-2 S protein coupled to ACE2. The scientists used AutoDock Vina to dock all FDA-approved drugs from two databases into the selected binding pocket of SARS-CoV-2-RBD and then rated them based on binding affinity. They evaluated the conformational changes by performing MD simulations of the compounds interacting with SARS-CoV-2-RBD in Gromacs version 5.1.2 for 100 ns. After heating the system to 300 K for 500 ps and keeping it in equilibrium using NVT ensembles, the simulation was run for 100 ns in 2-fs time steps, during which time the temperature was raised to 500 K again. The surface accessible surface area, polar solvation energy, van der Waals energy, binding free energy, and electrostatic energy were calculated using the MM-PBSA method [71].

The Schrodinger Suite protein synthesis wizard and the OPLS3e force field was used by Kanhed et al. [72] to improve the Mpro 3D structure from the Research Collaboratory for Structural Bioinformatics (RCSB) PDB. The SuperDRUG2-approved drug resource was used to access a 3-dimensional library of authorized drugs and diagnostic agents, and from there, the LigPrep module was used to synthesize 175,815 compounds. The Mpro protein structure was deposited in the PDB. A pharmacophore model based on the protein active site was built in Schrodinger’s Phase module using Glide XP scoring for the library of fragments. VS was performed using a seven-feature pharmacophore model. A pharmacophore model based on receptors was used to evaluate two different libraries. Two thousand two hundred fifty-three compounds passed the pharmacophore screening criteria. Using the pharmacophore-based filter, 26,001 compounds from the Asinex BioDesign library were accepted for the screen. For further inspection, only 15% most promising compounds were chosen. The selected compounds and Mpro were investigated via MD simulations carried out using the GROMACS 2020.1 software. The stability of ligand-receptor complexes was calculated using the CHARMM36 all-atom force field, whereas GROMACS was employed to establish ligand-receptor properties. Two 100-picosecond equilibration simulations were performed, the first using the conventional (NVT) ensemble and the second using the isobaric-isothermal (NPT) ensemble, after the energy of each complex had been reduced using the steepest descent approach [72].

M-Coffee, an MSA server extension of T-Coffee, and the HOMSTRAD, Prefab, and Balibase reference datasets were used for sequence similarity analysis. In the current study, 1,528 anti-HIV1 compounds are hand-picked for screening after being trained on compounds using a QFRET-based initial biochemical high throughput screening assay against 3CLpro of SARS-CoV 16. To create a more accurate prediction model, 2,382 chemical descriptors were mined from the complete compound library and run through the Weka program. Using the Auto Machine Learning (AutoML) method, the most efficient and clearly understandable ML algorithm was selected. Docking of the SARS-CoV-2 Mpro with the non-covalent inhibitor X77 was achieved via the GUI of version 0.8 of PyRx. The first screening was performed using a deep learning (DL) model utilizing the DL online server. GROMACS 18.7 was used to conduct MD simulations of the top-ranked poses of the screened complexes at a constant temperature of 300 K and pressure of 1 atm. A detailed investigation of the stability of the aquatic environment was carried out using the resultant trajectories [73].

Using the ChemoInfo VS program, which uses the docking method Protein-Ligand ANT System (PLANTS), a receptor-based VS (RBVS) was performed for each target protein. Drugs that affect the central nervous system were eliminated from the top 50 results that were obtained. The medicines were rated according to their ability to interact with multiple target proteins. Those with promising interactions with all three targets were put through MD simulation cycles and MM-PBSA calculations. Moldock Score, a docking method more precise than PLANTS, was used to submit medicines to MVD that demonstrated positive interactions with all three targets chosen for this experiment. To determine atomic charges, MD simulations were done using the OPLS-AA force field and the AcPype software, with the RESP approach set up to simulate the Hartree-Fock method. The ligands were entered into the GROMACS 5.1.4 program’s g_MM-PBSA tool to calculate the BFE, which followed the same procedure that was done in the prior study [31].

The UCSF Chimera tools to determine the crystal structure of SARS-CoV-2 Nsp16 were utilized by Jiang et al. [74]. Then they compared it to a Medications-lib of 7,173 stereoisomers representing 4,574 “licensed” medications using the MTiOpenScreen service. Using the information the authors provided, they searched the Probes and Drugs Portal to learn more about the targets and pathways of the compounds the authors were considering. MD simulations were used to re-create the physiological state of the protein represented by PDB entry 6W4H. The structures of proteins and protein-drug complexes were analyzed by computing various structural properties. They used MM-PBSA to estimate protein-drug binding affinity [74].

**Figure 2:**
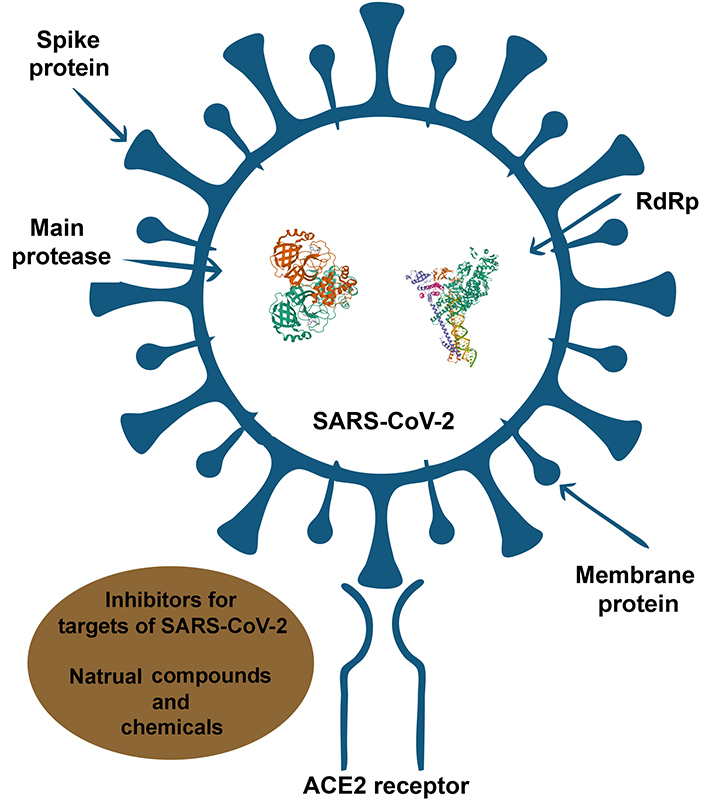
Potential mechanistic studies revealed different parts of SARS-CoV-2 and ACE2 receptor as targets for studied inhibitors

Finally, Table 2 demonstrates the obtained results of the literature studies on investigated compounds and COVID-19 targets in terms of their simulated affinities and mechanism of action. And, Figure 2 illustrates the mechanistic studies of SARS-CoV-2 target regions inhibited by various compounds.

**Table 2:** Mechanism of action for the available compound and their COVID-19-related targets

**Compound**	**Binding affinity** **(kcal/mol)**	**Target**	**Mechanism of action**	**Ref.**
ATV	WT-ATV –20.05 DLT-ATV –15.88 LMB-ATV –17.58	S-protein WT, delta, and lambda variant	Potent inhibitors targeting RBD of the S-protein	[45]
PRZ	WT-PRZ –17.81 DLT-PRZ –19.52	S-protein WT and delta variant		
14 Herbal isolates 10 Approved drugs	Below –8.10	S-protein	Inhibit the SARS-CoV-2 S-protein	[46]
28 Bioactive compounds	Below –5.60	Mpro or 3CLpro	Anti-SARS-CoV-2 activity	[47]
Saquinavir	–74.41	3CLpro	Inhibitors against the SARS-CoV-2	[48]
TCM5280805	–29.49			
Binifibrate	–69.04	Mpro	Bind strongly to the enzyme active site and hence they can be repurposed against SARS-CoV-2	[49]
Bamifylline	–63.19			
Simeprevir	–10.30	Mpro	Anti-SARS-CoV-2 activity	[50]
Ergotamine	–9.80			
Bromocriptine	–9.60			
Boceprevir	–13.60	Mpro	Inhibitors against the SARS-CoV-2	[2]
Epirubicin	–11.40			
Nelfinavir	–13.40			
Rutin	–14.90			
X77 “control”	–17.00			
Beclabuvir	–10.40	Mpro	Inhibit the replication of the virus	[75]
Nilotinib	–9.90			
Tirilazad	–9.60			
Trametinib	–9.50			
Glecaprevir	–9.40			
Hydroxychloroquine	Between –4.40 and –8.80	Multiple Mpros of SARS-CoV-2	Demonstrate efficacy against SARS-CoV-2	[52]
Chloroquine				
Remdesivir				
Lopinavir				
Ritonavir				
Umifenovir				
Favipiravir				
Epclusa (velpatasvir/sofosbuvir)	Between –9.00 and –10.10	Mpro	Very effective inhibitory actions on viral enzymes	[53]
Harvoni (ledipasvir/sofosbuvir)				
9 compounds	Between –6.05 and –11.23	S-protein (S-RBD)	Anti-SARS-CoV-2 activity	[54]
		ACE2		
Bedaquiline	-	Mpro	Inhibitors against the SARS-CoV-2	[76]
Glibenclamide				
Miconazole				
7 Drugs	-	Mpro	Inhibitors against the SARS-CoV-2	[55]
Vaborbactam	Below –5.40	Mpro	Anti-SARS-CoV-2 activity	[56]
Cimetidine				
Ixazomib				
Scopolamine				
Bicalutamide				
Riociguat				
9 Nucleoside triphosphate analogs	Below –8.70	RdRp	Anti-viral activity	[57]
Ibrutinib and zanubrutinib	Below –7.00	S-protein (S-RBD)	Inhibitors against the SARS-CoV-2	[58]
		Mpro		
		RdRp		
		ACE2		
		BTK		
		TMPRSS2		
		PLpro		
9 Currently approved molecules daunorubicin	Between –0.50 and –138.80	Mpro	Potential inhibitors of SARS-CoV-2.	[59]
Oxytetracycline	–9.30	3CLpro	Inhibitors against the SARS-CoV-2	[60]
Bemcentinib	–78.46	PLpro 3CLpro	Inhibitors against the SARS-CoV-2	[63]
Clofazimine	–165.60			
Abivertinib	–301.37			
Dasabuvir	–166.16			
MFCD00832476	–158.66			
Leuconicine F	–224.32			
Indinavir	Between –26.27 and –69.23	MPPs, spike ecto-domain, spike RBD, Nsp9 RNA binding protein, and HR2 domain	Fight SARS-CoV-2 infection	[64]
Sorivudine				
Cidofovir				
Darunavir				
Ledipasvir	Below –10.00	ACE2	Anti-SARS-CoV-2 activity	[30]
Estradiol benzoate		CD147		
Vancomycin		RdRp		
Paritaprevir and vancomycin		ACE2 and RdRp		
Lividomycin	–2,145.79	ACE2	Potential ACE2 inhibitors	[65]
Burixafor	–2,108.82			
Quisinostat	–1,998.77			
Fluprofylline	–1,785.00			
Pemetrexed	–1,602.58			
Spirofylline	–1,541.73			
Edotecarin	–1,312.19			
Diniprofylline	–1,292.42			
10 Molecules	Below –4.32	S1-RBD and the ACE2 receptor	Inhibitors of S-protein S1 domain and ACE2 interaction	[10]
Ribavirin	- (Relative docking Score below 2.80)	Mpro	Inhibitors of Mpro	[20]
Telbivudine				
Vitamin B12				
Nicotinamide				
Apixaban	-	Mpro	Anti-SARS-CoV-2 activity	[66]
Lapatinib	Approximately –30.00	3CLpro	Inhibit SARS-CoV-2 3CLpro	[67]
16 Potent inhibitors	Below –5.30	Mpro	Inhibitors of Mpro	[33]
Omipalisib/remdesivir	-	RdRp	Inhibitors against the SARS-CoV-2	[16]
Tipifarnib/omipalisib				
Tipifarnib/remdesivi				
Saquinavir	Below –6.00	Mpro	Inhibitors against the SARS-CoV-2	[70]
Beclabuvir				
Diammonium Glycyrrhizinate Digitoxin	–11.75 –11.25	S1-RBD and the ACE2 receptor	Inhibitors against the SARS-CoV-2	[71]
Ivermectin Rapamycin (Sirolimus)	–10.86 –10.56			
Rifaximin Amphotericin B	–10.54 –10.50			
Ritonavir	–8.01	Mpro	Mpro inhibitors	[72]
Nelfinavir	–7.62			
Saquinavir	–7.50			
Pralmorelin	–9.17			
Iodixanol	–15.77			
Iotrolan	–14.36			
Hesperidin	–10.30	Nsp16 2’-O-MTase	Potential inhibitors of 2’-O-ribose MTase	[73]
Rebastinib	–10.20			
Losulazine	–10.27			
Cep-32496	–10.20			
R428	–10.40			
Entrectinib	–10.57			
Osi-027	–10.50			
MK3207	–10.97			
Rimegepant	–10.77			
Bolazine	–10.47			
Paritaprevir, Simeprevir and Velpatasvir	Below –10.0	S-protein	Anti-SARS-CoV-2 activity	[61]
		Nucleocapsid protein		
		2’-O-ribose MTase		
Gonadorelin	–9.40	RBD-ACE2	Inhibiting the virus infection	[62]
Fondaparinux	–8.50			
Atorvastatin	–7.30			

Ref.: reference; WT: wild type; ATV: atovaquone; DLT: delta mutated RBD of S-protein; LMB: lambda mutated RBD of S-protein; PRZ: praziquantel; TMPRSS2: transmembrane serine protease 2; MPPs: main protease proteins; MTase: methyltransferase; -: blank cell

## Discussion

According to the most recent and cutting-edge approaches in cancer immunotherapy, antiviral CD8+ T cell immunity may be able to be used in cancer immunotherapies against various types of cancer. Infections or vaccines with SARS-CoV-2 cause this immunity [37].

Medical professionals and scientists have struggled to develop effective therapies for the deadly COVID-19 epidemic because of the massive loss of life it has caused. The repurposing of existing medications may fulfill the unmet need. The treatment of COVID-19 may be able to use many different anticancer medications in some capacity. The proliferative effects of these medicines are the primary focus of their development. Cytotoxic drugs and immune modulators can potentially suppress cancer patients’ humoral and cellular immunological responses, which may result in developing secondary infections and other problems. Patients hospitalized with COVID-19 should pay attention to titrating the medications to avoid over-suppression and further difficulties [38].

Patients with gastric cancer (GC) who are infected with COVID-19 can take one of the potential medications. There is a high binding affinity between these medicines and the Nsp12/RdRp region of SARS-CoV-2. After using docking to find drugs that would have a good chance of binding to the RdRp-NiRAN domain of SARS-CoV-2, four potent drugs with high binding affinities to the virus were selected. Due to the lack of a standard therapeutic option, medication repurposing is now viable for COVID-19 patients with GC. Patients with GC infected with SARS-CoV-2 may take advantage of these medications [39].

On the other hand, Pingali et al. [40] tested 27 medicines for treating small cell lung cancer (SCLC) for their ability to inhibit COVID-19’s RdRP protein and utilize the pharmaceuticals to treat both SCLC and COVID-19. For the RdRP complex, paclitaxel, gefitinib, and dacomitinib are projected to be suitable ligands [40].

Patients undergoing radiation or chemotherapy may benefit from Lamivudine since it reduces the risk of their Hepatitis virus becoming active again during treatment. Hepatocellular carcinoma (HCC) might be avoided in the first place if Lamivudine and similar ANNAs were effective in preventing chronic HBV and cirrhosis. Lamivudine, originally developed as a cancer treatment, may also be evaluated for its potential as a treatment for SARS-CoV-2 and COVID-19. Additional *in vitro* and *in vivo* testing of this chemical is required to determine whether or if it is effective against SARS-CoV-2 RdRp, either alone or in combination. Future theoretical and experimental research may benefit from the insights provided by this study. If these studies are successful, patients with cancer and COVID-19 will hopefully benefit from an improvement in their quality of life and their prognoses [41].

The SARS-CoV-2 S-protein was virtually screened for inhibitors using drug-likeness screening, molecular docking, and pharmacokinetic features evaluation. Researchers used molecular docking to zero in on the amino acid residues in ATV, PRZ, and binding sites responsible for the protein’s interaction with the spike. The WT-ATV and DLT-PRZ complexes were the most structurally stable over the simulation duration. In other words, the two medications were approved by the FDA after being shown to treat COVID-19 effectively. By shedding light on the underlying molecular mechanisms of inhibition, this work will significantly contribute to creating new SARS-CoV-2 inhibitors [45].

To stop SARS-CoV-2 from infecting human cells, technique of de Oliveira et al. [46] included looking at the virus’s S-protein before it connected with the human ACE2 receptors. Using binding affinities below –8.1 kcal/mol, they found anti-SARS activity in 14 herbal isolates and ten medicines. MD simulations show that the ligands for Lig8522, Lig8970, and Lig6843 were contained inside the RBD region for the simulations [46].

Medication repositioning was used in study of Tsuji [47] to locate potential medications that may be applied to treating SARS-CoV-2. These possible treatments might be subjected to further testing to evaluate whether or not they have anti-SARS-CoV-2 capabilities. The findings of these studies could be used to develop more specific anti-SARS-CoV-2 drugs [47].

The 3CLpro is a prospective therapeutic target for SARS-CoV-2 because of its central role in the viral replication cycle. In the current investigation, structure-based methods were used to repurpose anti-HIV pharmaceuticals and explore TCMs databases for compounds that may inhibit 3CLpro [48].

The residues of 3CLpro’s active site to those of databases of traditional Chinese medications, a structure-based method were compared by Arun et al. [49]. It is essential to conduct clinical trials of saquinavir and TCM5280805, two suspected inhibitors of 3CLpro, for the treatment of SARS-COV-2 [49].

It was revealed via computational and statistical techniques that Simeprevir, Ergotamine, Bromocriptine, and Tadalafil had the maximum binding affinity to the significant protease of SARS-CoV-2. Bromocriptine was also shown to have this affinity. There are many non-covalent interactions between these drugs and the residues in Mpro’s binding site [50].

Several existing medications, including rutin, boceprevir, epirubicin, nelfinavir, and bortezomib, show promise as inhibitors of SARS-CoV-2. These medicines are structurally similar to N3 and X77, two ligands cocrystallized with SARS-CoV-2 Mpro, and have identical binding patterns. This research found five promising COVID-19 treatments that extend the previously announced SARS-CoV-2 Mpro inhibitors [2].

D3Targets-2019-nCoV is a website the authors built for target prediction and VS using data on protein structures and ligands. The findings demonstrated that DL-based models are far better at finding COVID-19 hits than other approaches [51].

The sole current option for treating SARS-CoV-2 is the creation of pan-viral medicines based on highly conserved portions of existing medications. There were 129 medications evaluated for their potential usefulness in this research. There is evidence that the antiviral drugs beclabuvir, nilotinib, tirilazad, trametinib, and glecaprevir can stop the spread of COVID-19. To fully assess the efficacy of these putative inhibitors, however, more *in vivo* testing is required [75].

Compared with discovering new pharmaceuticals from scratch, the method of repurposing existing drugs for new therapeutic uses is more efficient in terms of the amount of time and the amount of money it saves, respectively. In some instances, the potential for acute toxicity from repurposed medications may be too great to justify the use of the drug in question as an antiviral, especially if the benefit is as yet unclear. As a result, an accurate evaluation of the probable candidates’ toxicity levels is required. *In silico* screening of FDA-approved drugs against SARS-CoV-2 proteins may help speed up clinical trials for drugs with a known safety profile [52].

A VS for commercially available drugs using the 3CLpro molecular model and provided 16 potential options for further study was conducted by Chen et al. [53]. Edipasvir and velpatasvir, two antiviral medications, stand out as promising treatments for the new CoV because of their low risk of causing the common but debilitating side effects—fatigue and headache. Epclusa (velpatasvir/sofosbuvir) and Harvoni (ledipasvir/sofosbuvir) are two combination drugs that have shown great promise as therapies for hepatitis C because of their ability to inhibit two separate viral enzymes [53].

According to the results of molecular docking assays, GR hydrochloride and GNF-5 predominantly link with hotspot 353, while RS504393, TNP, and Eptifibatide acetate engage with hotspot 31 via both polar and hydrophobic interactions. KT185’s affinity for hotspot 31’s S-RBD residue was predicted to block SARS-S-RBD. CoV-2’s. Some FDA-approved medications that were discovered through VS of compound libraries show promise as potential inhibitors of RNA viruses. These medications are thought to work by blocking viral entry or replication or reducing inflammation. The SARS-CoV virus evades the body’s defenses by binding to chemokine receptors. The inflammation caused by viral infection in cells and tissues may also be mitigated by these molecules [54].

Bedaquiline, glibenclamide, and miconazole, three medicines (two oral and one buccal), were shown to be effective Mpro inhibitors against COVID-19. These medications treat fungal infections, multi-drug resistant TB, and type 2 diabetes [76].

As part of the other authors’ efforts to streamline the drug development process, they have established two standardized procedures for repositioning studies that skip the time-consuming yet crucial pharmacokinetics assessment stage. Validation findings for these processes have been very positive, resulting in the selection of seven prospective medications that show encouraging signs of being effective against the CoV [55].

A combined structure-based VS technique may uncover possible irreversible Mpro inhibitors, which can be employed in creating antiviral medicines and potentially vaccinations using bioinformatics tools (FDA-approved drugs including vaborbactam, cimetidine, ixazomib, scopolamine, bicalutamide, and riociguat). Their study illustrates a combined cheminformatics process for identifying medications that can target diverse proteins of viruses [56].

Using drug repositioning, the authors of this research identified prospective anti-SARS-CoV-2 medication candidates and made predictions about therapies produced for the treatment of COVID-19. Results showed promise for the identification technique in rapidly repurposing medicines to manage COVID-19 [57].

In the pharmaceutical sector, production-linked incentive (PLI) research is crucial. Due to asymmetries in how these physicochemical characteristics are represented in the training dataset, the current techniques for predicting PLI suffer from lengthy compute times and biases. SSnet performs better on human and C. elegans datasets than other well-known ML algorithms. Additionally, it is much quicker than conventional VS methods. Accurate predictions of all three types of ligand binding sites (active, allosteric, and cryptic) are made using SSnet, which may be used with the classic VS/docking approach as a pre-screen to filter ligands. It is also easy for people of all levels of computer knowledge to use. Using their secondary structures as a common denominator, they proposed a latent space for proteins that may be utilized to make bioactivity comparisons [77].

Drugs already in use, such as ibrutinib and zanubrutinib, may be repurposed to help patients with COVID-19. They prevent viral polyproteins from being assembled, replicated, and post-translationally processed, all of which are a part of the inflammatory cytokine storm mediated by BTK [58].

**Figure 3:**
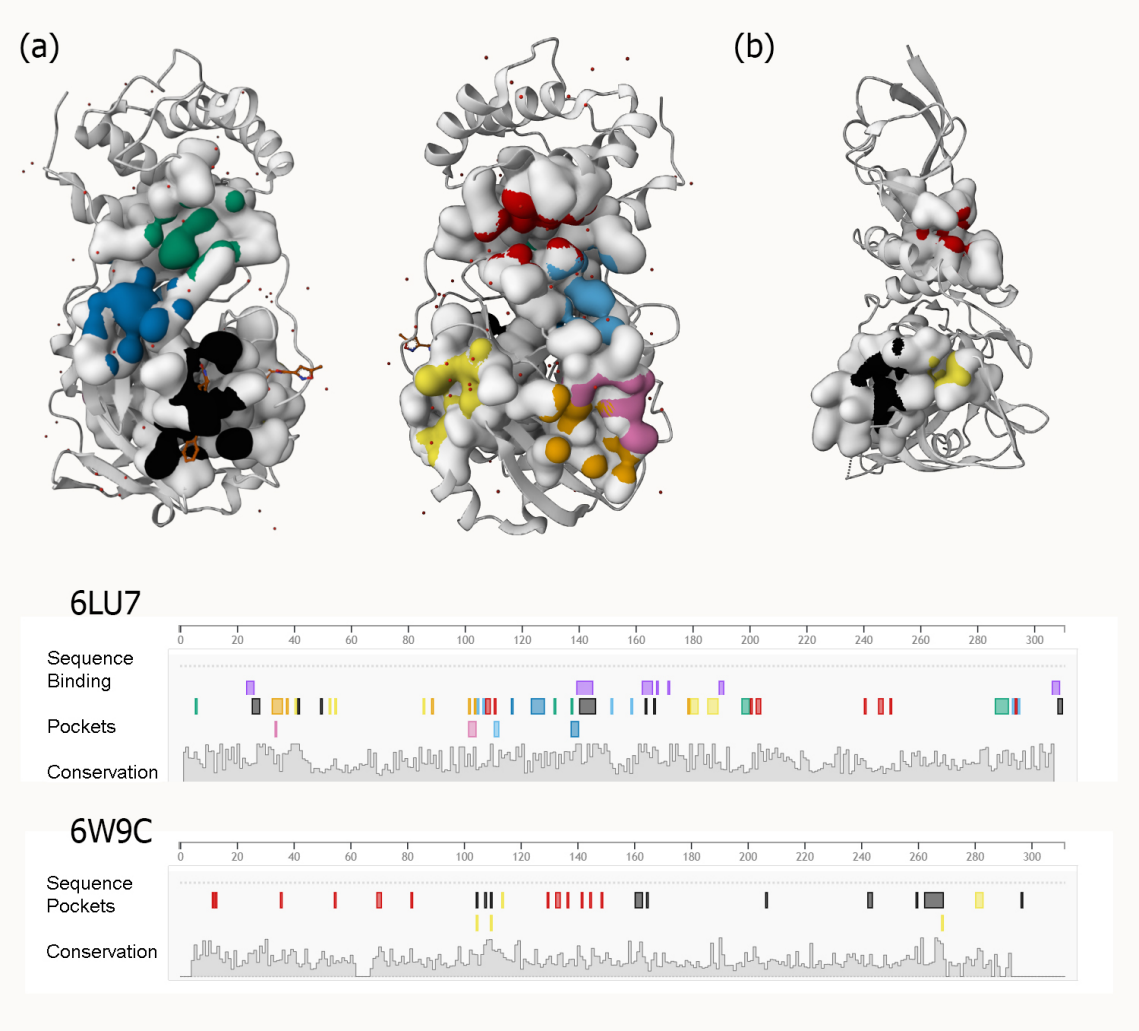
Examples of predicted active sites for two viral proteins [(a) PLpro and (b) 3CLpro] with PDB IDs 6W9C and 6LU7, respectively

To construct medications rationally, it is necessary to consider the high degree of flexibility present in the active site of the SARS-primary CoV-2 protease (potent active sites are shown in Figure 3). Using three different Mpro conformers, Jiménez-approach Alberto was able to isolate nine molecules that might potentially be repurposed as a treatment for COVID-19 [59].

This research has led to the identification of several therapeutic strategies that offer promise for combating the SARS-CoV-2 pandemic. Oxytetracycline, naringin, kanamycin, cefpiramide, salvianolic acid b, teniposide, etoposide, and DOX were the hits that showed the most promise in the fight against cancer. After that, the researchers screened the chemical space using a pharmacophore search, and they found a variety of novel scaffolds. Some examples of these new scaffolds are the fluoroquinolone ZINC001570001158 and the sulfonamide ZINC000016429284. In addition, the findings of the research point to the possibility that antibiotics that are commercially available right now, most notably oxytetracycline, may have a substantial impact on the ability of SARS-CoV-2 to create 3CLpro [60].

The supercomputer MOGON also provided some ideas for potential inhibitors of the SARS-CoV-2 virus. The therapeutic potential for SARS-CoV-2 includes several medicines that are currently on the market for illnesses caused by encapsulated ssRNA viruses, such as cancer, HCV, and other diseases [61].

The RBD of SARS-CoV-2 S-protein and human ACE targets current medications that have been identified via computational studies of drug repurposing (ACE2). An MD simulation of RBD produced an ensemble of structures. In the VS process, three significant conformers were found and analyzed [62].

Researchers ran an *in silico* high throughput screening on 5,491 compounds from multiple databases to find SARS-CoV-2 proteases PLpro and 3CLpro inhibitors that might treat COVID-19. Ergotamine and abivertinib are good drug development candidates for COVID-19 treatment [63].

Numerous physiologically active structural analogs from DrugBank, including the antiviral medicines indinavir, sofosbuvir, cidofovir, and darunavir, have shown promise in fighting SARS-CoV-2 infection [64].

Medication repurposing using VS was used to track down CD147 inhibitors, ACE2 inhibitors, and RdRp inhibitors for this study. Based on the data, the best g-values were found for the combinations of ledipasvir and ACE2, estradiol benzoate and CD147, and vancomycin and RdRp. Significant viral receptor inhibition can stop COVID-19 infection. Possible choices for more study included paritaprevir and vancomycin [30].

A chemical library of several thousand human-licensed medicines using MM-assisted SBVS was searched by Teralı et al. [65] to uncover drugs that might interact with human ACE2 and prevent SARS-identification CoV-2’s. All or part of the eight found medications have proven *in vitro* and *ex vivo* effectiveness against COVID-19. Hence, they should be brought into the clinics as monotherapy or in conjunction with other treatments [65].

Fenoterol and riboflavin ligands on grid 1 (site S1), Cangrelor on grid 2 (site 2), and Vidarabine on grid 4 (sites 1 + 2) all exhibited intense contact with the RBD area. These potential inhibitors of S1:ACE2 need to be investigated *in vitro* to determine whether or not they are effective [10].

The Mpro of the recently identified COVID-19 was compared to the Mpro of SARS and MERS CoV, which were two former CoV pandemics. These comparisons were made using molecular modeling techniques. COVID-19 Mpro has a stronger genetic relationship to SARS Mpro than vice versa. In a VS competition against COVID-19 Mpro, it was shown that curcumin had the lowest level of effectiveness when compared to a panel of antivirals, vitamins, anti-microbial, and other systemically acting drugs (known Mpro inhibitors). Alternative applications are suggested for vitamin B12, nicotinamide, and the nucleoside reverse transcriptase inhibitors ribavirin and telbivudine [20].

As an alternative therapy method for COVID-19, Hage-Melim suggests new possible Mpro inhibitors of the SARS-CoV-2 virus in this study. These compounds’ ADME/Tox characteristics were superior to those of the medicines being evaluated against COVID-19, and they maintained a network of favorable intermolecular interactions with the novel CoV. For the future therapy of patients with this SARS, the authors recommend their most promising chemical in addition to Apixaban [66].

Another study effectively used a mix of molecular docking-based computational screening and *in vitro* testing of repurposed medicines to identify compounds that inhibit SAR-CoV-2 3CLpro. The level of 3CLpro inhibition shown by lapatinib was the greatest of any drug. Van der Waals interactions primarily drove the binding of lapatinib to 3CLpro. Some of the drug’s stability comes from hydrogen bonds formed with the N142 and E166 residues. To create effective SAR-CoV-2 3CLpro inhibitors, it is best to use both *in silico* screening and experimental research [67].

Due to its importance in viral replication, the SARS-CoV-2 Mpro might be a therapeutic target. The perfect candidate would be substantial enough to fill the pocket. They employed a hybrid strategy using VS and molecular docking methods to search through a collection of natural chemicals and structural mimics identified or synthesized by the Mazzini team. To enter the protein binding pocket and access the catalytic dyad, the most promising candidates have shown impressive abilities as potential scaffolding for creating superior inhibitors. CPT, Leopolis acid, and lamellarin D derivatives are desirable options. To develop effective SAR-CoV-2 3CLpro inhibitors, it is best to use both *in silico* screening and experimental research [68].

The predictive capacities of the provided docking approaches were assessed using a database of 500 compounds previously established as potent inhibitors of the SARS-CoV 3CLpro enzyme. For each docking simulation, the authors generated numerous poses for each ligand and considered all potential isomers/states for molecules that may exist in more than one configuration. Consensus models were built after calculating the resultant binding and isomeric space. The top-ranked compounds include a range of heterogeneous molecules, many of which have recently been shown to inhibit SARS-CoV-2 3CLpro. The findings encourage the repetition of comparable docking techniques by simultaneously considering both monomers of various representative resolved structures. Despite the significant conservation shared by SARS-CoV 3CLpro and genuine inhibitors, current prediction models benefit from using known inhibitors. Post-docking instruments utilized to dock SARS-CoV-2 3CLpro successfully had significantly varying roles, but they all helped get the job done [69].

Using the FEP-ABFE prediction method, Li et al. [33] could isolate 16 potent inhibitors of SARS-CoV-2 Mpro from currently available drugs. The efficiency of 14 of these inhibitors was confirmed. Clinical studies are underway for DIP, the most potent inhibitor presently known. Newer drugs that reduce SARS-CoV-2 Mpro include candesartan cilexetil, hydroxychloroquine, and chloroquine. In the future, these drugs might be used as templates for creating treatment options with increased effectiveness against SARS-CoV-2 Mpro. With a stunning 60% hit rate, FEP-ABFE prediction-based VS has shown to be very effective [33].

Jang found three promising SARS-CoV-2-inhibiting drug combinations at therapeutic dosages. Tipifarnib and Omipalisib, Tipifarnib and Remdesivir, and Omipalisib and Remdesivir, respectively, are the medication combinations in question. Based on promising results from SARS-CoV-2 inhibition testing in cells, the authors advise moving on with preclinical and clinical trials using omipalisib and three-drug combinations [16].

Medications with untapped therapeutic potential may be found via computational analyses—patients at an increased risk for contracting COVID-19 due to cardiovascular diseases. The data may be used to choose medications for *in vitro* and *in vivo* investigations, with Saquinavir and Beclabuvir being the best new protease inhibitor options for COVID-19 treatment [70].

Many promising lead compounds were identified by using the SARS-CoV-2-RBD crystal structure with a VS of clinically approved drugs. Furthermore, the binding free energy of the SARS-CoV-2-RBD complex was lowest for the Diammonium Glycyrrhizinate complex [71].

For the treatment of SARS-CoV-2, three antiviral medicines were selected from the currently available drug library. Three diagnostic compounds were identified by screening an authorized drug library that showed promise in interacting with the Mpro protein; structural alterations to these three candidates might lead to valuable molecules. Twenty compounds were found to interact with the viral Mpro protein by a rigorous VS of the Asinex BioDesign library [72].

Compounds found in Cluster Two were tested for hits against 3CLpro, and two were re-docked with four distinct PDB structures of 3CLpro for further analysis [73].

An exhaustive theoretical investigation was carried out by us, in which the authors used the approaches of RBVS, docking, MD simulations, and MM-PBSA computations. In addition to Remdesivir, our seven possible hits revealed the ability to bind to three of the most critical SARS-CoV2 targets efficiently. Remdesivir, elbasvir, and ritonavir are the most promising medications for this purpose, as shown by our findings [31].

**Figure 4:**
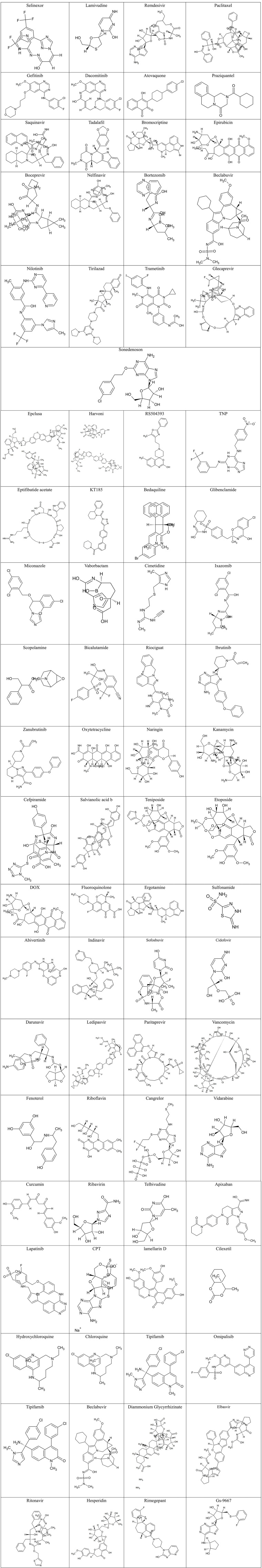
Structures of reviewed final compounds obtained from the virtual screening procedures

SARS-2’-O-MTase CoV-2’s Nsp16, which causes COVID-19, structurally and functionally was examined by Jiang et al. [74]. Inhibitors of 2’-O-MTase have been found in 17 different drugs, several of which are currently in clinical use. Further experimental and/or clinical testing of hesperidin, rimegepant, gs-9667, and sonedenoson, among other potential possibilities for treating COVID-19, is warranted [74]. Finally, the extracted chemicals from several studies are listed in Figure 4.

Taking all information mentioned above together into consideration, the target of evaluation for a ligand capable of inhibiting a receptor specifically for various types of SARS-CoV2 viral proteins was only determined by calculations of energy binding affinity values using either molecular docking scores (e.g., AutoDock Vina, iDock, AutoDock, DockThor, and Glide) or MD simulation denoted by G (e.g., MM-PBSA and MM-GBSA methodologies). However, no experimental values were reported on confirmation of the calculated values.

Due to hardware limitations or a lack of target proteins, previously unavailable approaches are now widely available in the in-silico docking field [78]. Researchers are increasingly interested in this topic because of the PDB’s large collection of macromolecules and its fast-advancing hardware components. As pharmaceutical companies increasingly use high-throughput screening (HTS) for discovering new, innovative drug substances, a more economical alternative is needed. The *in silico* equivalent of HTS, VS, allows you to test a large number of compounds in a short amount of time. Even though some programs are capable of generating poses similar to those found in crystal structures, protein-ligand docking has several limitations. In most docking methods, proteins are considered rigid for practical reasons, while ligands and proteins are treated as flexible [79, 80]. Further, unlike what we might expect, the structure of ligands can greatly influence docking scores and pose scores [81]. The docked poses can therefore be drastically affected by even minor changes in ligand input conformation [81]. The scoring functions of docking programs have a major limitation, as they do not always provide accurate predictions of ligand affinity to substrates [82, 83]. Furthermore, molecular docking has the disadvantage that water molecules do not enter binding sites, so the ligand’s interaction with the target can be inaccurate. Docking programs may be made more effective by combining them with other techniques. It is possible to estimate ligand-binding affinities with just a moderate amount of computational work using a variety of well-established approaches, such as standard MD or binding free energy estimates like MM-GBSA and MM-PBSA [84].

It is also acknowledged that commercially targeted or focused libraries have evolved well beyond COVID-19, a topic that is beyond the scope of this paper. To aid in the preliminary VS stages enabled by these libraries, commercial and other suppliers of focused and targeted libraries are required. When using targeted libraries, readers should be cautious about compound selection, which is key to success in drug discovery efforts.

## Conclusions

Little research has been done to determine the exact nature of the connections between COVID-19 and cancer. Studies are being conducted to determine whether or not cancer patients would have more severe COVID-19 symptoms and whether or not the likelihood of developing COVID-19 will rise as a result of cancer therapies. There is some indication that specific cancer therapies may be beneficial against the COVID-19 virus. Despite this, there is mounting evidence that pharmacological repurposing and VS might play significant roles in the battle against COVID-19. VS and the repurposing of existing cancer drugs might be helpful in searching for COVID-19 therapies. The SARS-CoV-2 virus has several distinctive characteristics that set it apart from other viruses and make it an intriguing target for medication repurposing and VS. The first is that the structure of the viral S-protein is quite similar to the structure of other well-known human viruses, such as the virus that causes the common cold. Based on this, antiviral medications now in use against other human viruses may also be effective against SARS-CoV-2. When an existing medicine is repurposed, it is used for a different purpose than it was initially intended. This strategy may be pursued for both approved medications and those tested in humans but found to be ineffective for other uses. The computer method known as VS may be used to evaluate many compounds to determine which ones have the most significant potential to form bonds.

## Abbreviations

3CLpro: 3-chymotrypsin-like protease
3D: three-dimensional
ACE2: angiotensin-converting enzyme two
ANNAs: antiretroviral nucleoside/nucleotide analogs
ATV: atovaquone
BFE: binding free energies
BTK: Bruton tyrosine kinase
CoV: coronavirus
COVID-19: coronavirus disease 2019
DL: deep learning
DLT: delta mutated receptor binding domain of spike protein
DOX: doxorubicin
FDA: Food and Drug Administration
FEP-ABFE: free energy perturbation-absolute binding free energy
GC: gastric cancer
GROMACS: Groningen MAchine for Chemical Simulation
HIV: human immunodeficiency virus
MD: molecular dynamics
MM-GBSA: molecular mechanics with generalised born and surface area
MM-PBSA: molecular mechanics Poisson-Boltzmann surface area
Mpro: main protease
MTase: methyltransferase
NiRAN: associated nucleotidyl transferase domain
Nsps: non-structural proteins
PDB: Protein Data Bank
P-gp: P-glycoproteins
PLpro: papain-like protease
PRZ: praziquantel
RBD: receptor binding domain
RdRp: RNA-dependent RNA polymerase
SARS: severe acute respiratory syndrome
SARS-CoV-2: severe acute respiratory syndrome coronavirus 2
SCLC: small cell lung cancer
S-protein: spike protein
S-RBD: spike protein receptor binding domain
TCM: traditional Chinese medicine
VS: virtual screening
WT: wild type

## Declarations

### Copyright

© The Author(s) 2023.
